# Exploring barriers and facilitators of physical distancing in the context of the COVID-19 pandemic: a qualitative interview study

**DOI:** 10.12688/hrbopenres.13295.2

**Published:** 2021-07-20

**Authors:** Karen Farrell, Hannah Durand, Jenny McSharry, Oonagh Meade, The Health Psychology Public Advisory Panel, Eanna Kenny, Chris Noone, Laura L. O'Connor, Kim L. Lavoie, Molly Byrne, Robert Mooney, Brian E. McGuire, Gerard J. Molloy

**Affiliations:** 1 School of Psychology, National University of Ireland, Galway, Galway, H91 EV56, Ireland; 2 Montreal Behavioural Medicine Centre, Centres intégrées universitaire de santé et de services sociaux du Nord-de-l’Île-de-Montréal (CIUSSS-NIM), Montreal, Quebec, QC H4J 1C5, Canada; 3 Department of Psychology, University of Quebec at Montreal, Montreal, Quebec, QC H2L 2C4, Canada; 4 Communications, Department of Health, Government of Ireland, Dublin, D02 XW14, Ireland

**Keywords:** Physical distancing, COVID-19, qualitative, barriers and facilitators, PPI

## Abstract

**Background: **Physical distancing measures (e.g., keeping a distance of two metres from others, avoiding crowded areas, and reducing the number of close physical contacts) continue to be among the most important preventative measures used to reduce the transmission of severe acute respiratory syndrome coronavirus 2 (SARS-CoV-2) that causes coronavirus disease 2019 (COVID-19). Therefore, it is important to understand barriers and facilitators of physical distancing to help inform future public health campaigns.

**Methods: **The current study aimed to qualitatively explore barriers and facilitators of physical distancing in the context of the COVID-19 pandemic using a qualitative interpretative design. Semi-structured one-to-one phone interviews were conducted with 25 participants aged 18+ years and living in the Republic of Ireland between September and October 2020. A purposive sampling strategy was used to maximise diversity in terms of age, gender, and socioeconomic status. Interviews were analysed using inductive thematic analysis.

**Results: **Analysis resulted in the development of six main themes related to barriers and facilitators of physical distancing: (1) Maintaining and negotiating close relationships; (2) Public environments support or discourage physical distancing; (3) Habituation to threat; (4) Taking risks to protect well-being; (5) Personal responsibility to control the “controllables”; and (6) Confusion and uncertainty around government guidelines.

**Conclusions: **Physical distancing measures were judged to be more or less difficult based on a number of internal and external psychosocial factors. Barriers to distancing included difficulties maintaining and negotiating close relationships, habituation to COVID-19-related threat, risk compensation, and confusion and uncertainty around government guidelines. Having a sense of personal responsibility to prevent COVID-19 transmission through distancing was an important facilitator. The structure of public environments was viewed as both barrier and facilitator. Barriers and facilitators may vary depending on context and life stage, which should be considered in the design of interventions to target physical distancing behaviour.

## Introduction

Physical distancing measures continue to be among the most effective preventative measures used to curb the transmission of severe acute respiratory syndrome coronavirus 2 (SARS-CoV-2) that causes coronavirus disease 2019 (COVID-19) ( Anderson *et al.*, 2020; Islam *et al.*, 2020). While the development and widespread availability of efficacious vaccinations to manage the COVID-19 pandemic may turn out to be the most impactful scientific and public health achievements of recent times, public health behaviours, including physical distancing, remain essential ( Moore *et al.*, 2021). Until most of the global population are vaccinated and the subsequent epidemiological data show that COVID-19 morbidity and mortality is reduced to a manageable level for local health services, physical distancing will be a primary means of saving lives and maintaining public health.

Physical distancing is a set of measures intended to prevent the spread of a contagious disease by maintaining a physical space between people and reducing both the number of times people come into close physical contact with one another and the duration of contact ( Harris *et al.*, 2020). These mitigation measures include keeping a distance of two metres from others, limiting household visitors, reducing number of contacts, avoiding crowded places, avoiding non-essential travel, working from home where possible, and only leaving home for essential reasons and exercise. For the elderly and those with pre-existing medical conditions, more stringent measures are advised to reduce the risk of contracting COVID-19, such as staying at home and limiting face to face communication with other people as much as possible ( Health Service Executive, 2020).

Evidence gathered during the first 12 months of the pandemic has indicated high and sustained self-reported levels of physical distancing in many countries. This is evident from Irish public opinion data reported in December 2020, which shows consistently high self-reported frequency of adherence to public health recommendations to prevent community transmission of COVID-19 from July to December 2020 ( Amárach Research, 2020). Data gathered in Ireland in June 2020 as part of the International COVID-19 Awareness and Responses Evaluation (iCARE) study also showed that adherence was high for most physical distancing measures, with maintaining 2-metres between individuals being the physical distancing behaviour with the highest overall level of adherence ( Durand *et al.*, 2021). Similarly, high levels have been seen internationally ( Beeckman *et al.*, 2020; Coroiu *et al.*, 2020; Pfattheicher *et al.*, 2020). Despite the overall positive findings in relation to adoption of physical distancing behaviours, the spikes in transmission and subsequent burden on healthcare at several key points during 2020 suggest that adequate physical distancing in Ireland did not occur, particularly following the relaxation of government restrictions in the later part of 2020. There are a complex range of inter-related individual, emotional, family, community, societal, political, and cultural factors that may be relevant in understanding this (e.g., Atchison *et al.*, 2020; Durand *et al.*, 2021; Williams *et al.*, 2020; Wirz *et al.*, 2020). Therefore, qualitative evidence can play an important role in describing the challenges of maintaining physical distancing from the perspective of the lived experience of members of the public.

The present study aimed to explore barriers and facilitators of adherence to various physical distancing measures in the context of the COVID-19 pandemic.

## Method

The current study is reported in line with the COnsolidated criteria for REporting Qualitative research (COREQ) checklist ( Tong *et al.*, 2007). A protocol for this study has been published ( Durand *et al.*, 2020a). The current study forms part of a broader project focused on physical distancing in Ireland, which is registered with the Open Science Framework ( Durand *et al.*, 2020b). Our PPI processes are reported in line with the Guidance for Reporting Involvement of Patients and the Public Version 2 (GRIPP2) checklist ( Staniszewska *et al.*, 2017).

### Aim 

The aim of this study was to qualitatively explore barriers and facilitators of physical distancing in the context of the COVID-19 pandemic.

### Ethical approval

Ethical approval was sought and granted for this work by the Research Ethics Committee at the National University of Ireland (NUI), Galway (Ref no.: HRB20-Apr-18).

### Study design

The study employed a qualitative interpretative design.

### Sample selection and recruitment

Participants were adults aged 18 years or older who were resident in Ireland from September to October 2020. A purposive sampling strategy, which involved choosing participants deliberately based on the specific qualities they possess ( Etikan *et al.*, 2016), was employed. For this study, participants were selected to ensure the sample was diverse in terms of age, gender, and occupational category (e.g., professional/managerial, technical, service and sales worker, student, casual worker, unemployed, retired, etc.).

Recruitment for the study was publicised through targeted social media advertisements via Facebook, Twitter and Instagram platforms and email advertisements circulated via community groups, professional organisations, and personal networks. All participants who expressed interest were directed to a Google Forms link by a member of the research team and asked to fill out demographic and contact information. Participants were then selected using a sampling matrix to ensure maximum variation. Selected participants were given the information leaflet to review, and the interview date and time was scheduled via email. Participants were offered a multi-store gift card to the value of €25 for their participation in the study. 

### Sample size

A total of 42 individuals expressed interest in participating via email. Of these, 25 were recruited to participate. This aligns with published recommendations of 10–20 participants for qualitative studies utilising thematic analysis approaches ( Braun & Clarke, 2013). These participants were selected based on their fit within the purposive sampling matrix. The remaining 17 people who expressed interest were respectfully declined and thanked for their interest in the study. Due to resource constraints, it was not possible or feasible to interview everyone who expressed interest. Initial data analysis took place alongside data collection and interviews continued until data was collected that was adequate in both amount and variety to answer the research question ( Vasileiou *et al.*, 2018).

### Data collection

Participants were presented with the option to participate in a one-to-one semi-structured interview via phone or a secure web-based platform (e.g., Microsoft Teams); all elected to complete the interview via phone. A topic guide of open-ended questions was used to flexibly guide the interviews (see Extended data; Durand *et al.*, 2020b). In brief, the topic guide addressed participants’ personal experience of COVID-19, what they understood by the term ‘physical distancing,’ perceived barriers and facilitators of physical distancing in public and in private, and their perceptions of government communications around physical distancing. Non-directive prompts were used to elicit further detail as needed. Participants were also asked if they wanted to say anything else they felt was relevant that the topic guide did not address. The topic guide was developed by the research team and a panel of seven members of the public (the contribution of the public and patient involvement [PPI] panel is described in detail below) from an initial review of existing literature and personal experience of engaging in physical distancing behaviours. The topic guide underwent several stages of refinement with input from the PPI panel. Informed consent was sought and obtained via audio recording from all participants prior to the interview. The interviews were conducted over four weeks from the 28 ^th^ of September 2020 to 22 ^nd^ of October 2020 and lasted between 30–45 minutes. Interviews were audio-recorded; interview recordings were stored in a separate secure location from the audio-recorded statements of informed consent to ensure participants could not be easily identified. Interviews were conducted and transcribed verbatim by a member of the research team who had practical skills and experience in conducting qualitative research (KF; Patton, 1990). Field notes were taken to record the researcher’s initial observations about the interview data and to aid in future analyses. In the interest of time, given the urgency of COVID-19 research, transcripts were not returned to participants for comment or correction.

### Context

From September 28 ^th^ to October 22 ^nd^ the national cumulative incidence of confirmed cases per 100,000 of the population rose from 734.73 to 1,121.62 (see daily infographic of the Epidemiology of COVID-19 in Ireland from the Health Protection Surveillance Centre (HPSC) website). At this time, restrictions in Ireland were between Level 2 (all counties except Dublin and Donegal until 6 ^th^ October), Level 3 (Dublin and Donegal) and Level 4 (Cavan, Monaghan, and Donegal) with a move to Level 5 (lockdown 2.0) for the whole country on 21 ^st^ of October 2020, when the number of confirmed cases reached the highest recorded to that of the first wave in April 2020. An explanation of the restrictions in place in Ireland at each Level from Level 1 (least restrictive) to Level 5 (most restrictive) Government of Ireland's plan for living with COVID-19. In brief, in counties at Level 2, most areas of economy and society (e.g., schools, restaurants, pubs, and gyms) were open with protective measures in place (e.g., physical distancing, mandatory face coverings, increased facilities for hand hygiene and cleaning of surfaces), and social gatherings of a maximum of six people from four households were allowed in homes and gardens or in public. At Level 3, some businesses were closed (e.g., museums, indoor cultural venues), restaurants and pubs were open with additional restrictions relative to Level 2, and social gatherings were restricted to a maximum of six people from two households in public settings only. At Level 4, only essential businesses and services were open and no social gatherings were allowed. 

### Patient and public involvement

A PPI group was involved in the design, recruitment, topic guide development, and analysis stages of the study. PPI panel members were recruited through PPI Ignite at NUI Galway, through existing PPI groups, and through advertisements to NUI Galway Students’ Union, and NUI Galway University of Sanctuary. Two to three members of the research team (OM, HD, and KF) met on four occasions with PPI partners online via the Zoom video communications platform. The purpose of these meetings was firstly to review the interview topic guide for its clarity and relevance and to give advice on strategies to recruit a diverse sample. PPI members also contributed to the qualitative analysis in a three-step process: Step 1: Contributors were given a brief introduction to qualitative research and coding. We then sought their assistance in inductively coding a sample of passages from the interview transcripts. This was done to ensure the team had not missed any important barriers/facilitators and to feed into the development of the thematic map. Step 2: PPI members were involved in validating the themes identified, by reviewing the list of themes and corresponding quotes and commenting on whether the themes made sense in relation to the research question, and if they felt the codes provided compelling evidence for each theme. Step 3: This meeting was to discuss contributor’s feedback on the draft analysis section. The PPI contributors also gave additional time between formal meetings to review the thematic map and analysis section of this article. Panel members played a fundamental role in the research process by helping to shape the design and analysis, enhancing the quality and appropriateness of this research. Members of the PPI panel received a multi-store gift card to the value of €25 for each of the four meetings they attended and / or for additional time spent reviewing written material. This is in line with the UK National Institute for Health Research (NIHR) Central Commissioning Facility policy on the payment of fees and expenses to members of the public ( NIHR, 2018).

### Data analysis

Reflexive thematic analysis was used to analyse interview transcripts under the guidelines described by Braun & Clarke (2006). This analytic approach was chosen as it is highly flexible and provides a rich and detailed, yet suitably complex account of qualitative data ( Braun & Clarke, 2019; King, 2004). The data was managed using NVivo 12 Software ( QSR International, 1999). An inductive approach to qualitative data analysis was taken, such that themes were identified solely from the data. Each transcript was read independently, and initial codes were assigned to the data by KF. These codes were shared with a member of the research team (HD) and refined through discussion. Initial candidate themes were identified from the codes and discussed with other members of the research team (KF, HD, OM, JMS, GJM). All team members had a background in psychology and interest in health behaviour. These themes were further developed and refined in a virtual reflexive group session. PPI members also contributed to the qualitative analysis as described above.

## Results

A total of 25 participants (14 female) were interviewed via phone. Participants interviewed were of varying age (18–80 years) and occupational status. Most participants (n = 14) were able to work from home at least some of the time. Table 1 summarises participant characteristics and illustrates the variety in the sample.

**Table 1.** Participant characteristics.

Characteristic	*n*(%)
**Gender**	
Female	14 (56%)
Male	10 (40%)
Agender	1 (4%)
**Age Range**	
18–24	12 (48%)
25–34	6 (24%)
35–44	1 (4%)
44+	6 (24%)
**Occupational Classification**	
Professional/Managerial	7 (28%)
Student	7 (28%)
Casual worker	5 (20%)
Unemployed	2 (8%)
Unemployed due to long term sickness	1 (4%)
Retired	3 (12%)

Six themes related to potential barriers and facilitators to physical distancing were developed: (1) Maintaining and negotiating close relationships; (2) Public environments support or discourage physical distancing; (3) Habituation to threat; (4) Taking risks to protect maintain well-being; (5) Personal responsibility to control the “controllables”; and (6) Confusion and uncertainty around government guidelines. The six themes are described below with quotations provided as typical examples of theme content, to explore the differences between participants or to highlight issues of particular interest. Demographic information is provided where relevant or when explicitly described by participants during their interviews.

### Maintaining and negotiating close relationships

This was a central theme identified in the analysis with all participants describing maintaining and negotiating close relationships as the most difficult behaviour and a barrier to physical distancing. Two subthemes identified within this theme were: (1) Difficulties of physical distancing and sacrificing human contact and (2) Having to negotiate distancing with others.

***Difficulties of physical distancing and sacrificing human contact***

Many participants described the difficulty of being unable to see or having to maintain distance with family members and close friends outside of their household, and how challenging and abnormal this felt.

“ *I think because our normal actions and our normal interactions with other people like previous to this for however long we're on this planet, would have been social, would have involved touching, and hugging, and also being close to somebody not just speaking with them or having a good time with them, you just don't forget that. It's almost like a motor reaction, where you're kind of putting yourself into a situation where you're not necessarily physically distancing with each other,”* (Male, 28, professional/managerial).

Loneliness and feelings of missing out on the college experience made physical distancing particularly challenging for students.

*“I definitely think it's a lot harder being back in college because obviously you want to see your friends, it's my last year as well so it's a bit sad. So, it's definitely harder I think with people my own age, yeah just friends and stuff that you miss,”* (Female, 23, student)

***Having to negotiate distancing with others***

Negotiating distancing with friends and family members was a barrier to physical distancing where participants felt unable to refuse physical affection or closeness from loved ones. There was a sense of worry prior to interacting with friends and family that they would not be as cautious or choose to maintain distance. A female participant explains the difficulty of maintaining distance from an elderly family member when they came to visit after the first lockdown.

“…when he [grandad] came down like I had my mask on, I was still very, very scared of everything at that point. He just had cancer last year, so I was very worried about him, and as soon as I opened my house door he just came and hugged me immediately there was no social distancing and you just can't say no, you know? I couldn't stay ‘You can't do that grandad,’ and then the rest of the trip I kind of just thought oh well f**k it, it's too late now like we've already hugged. So yeah, the rest of the trip we didn't socially distance with each other,” (Female, 26, unemployed).

Participants expressed ambivalence around negotiating distancing with others, whereby they believed distancing was important but were concerned about potential negative responses from other people. The worry and anxiety of having to negotiate distancing with friends, being nervous to ask friends to keep a two-metre space when meeting up in social settings, in fear that it will instigate feelings of awkwardness and embarrassment and that others will not feel the same obligation or concern to maintain physical distancing made it difficult for participants to continue distancing.

*“…there's kind of two, maybe three [friends] that I'd meet up with on a regular basis, and I do find social distancing with them quite difficult, because I always worry like if I said ‘oh can you keep your two metres’ that there just going to kind of laugh at me and be like what are you doing that for, don't be ridiculous,”* (female, 26, unemployed).

### Public environments support or discourage physical distancing

The physical environment and safety measures in place in retail and recreational settings were important barriers and facilitators to physical distancing, for both patrons and staff. Many participants discussed how comfortable or uncomfortable they felt when in retail and recreational environments such as supermarkets, restaurants, and gastro pubs. There were mixed opinions on the safety measures taken in these settings, where participants felt adherence to physical distancing was made easier or harder based on the set up of different restaurants or shops.

One participant explained how they felt so uncomfortable in their local shop that they decided to leave due to the lack of preventative health measures taken by management and staff to protect customers when grocery shopping. They described witnessing other customers in the shop not wearing a mask when masks were mandatory at that time and failing to physically distance from each other, or practice appropriate coughing and sneezing etiquette.

*“I did leave a shop in my hometown. There were no controls in place, people weren't wearing masks. I remember just having this kind of you know feeling of, god, I've never felt so uncomfortable in my local shop. I think within two minutes I said, ‘right, we're just leaving right now I'm not staying here.’ It was just there was no physical distancing, people were standing up right beside you and just super, super uncomfortable,”* (Male, 25, student).

Positive experiences were also encountered, for example, going to a restaurant for a friend’s birthday. A female participant explained how she felt quite nervous before attending, wondering what measures the establishment would have in place, but described feeling safe and comfortable as a result of the guidelines enforced by management and staff to facilitate physical distancing.

*“I remember the first time we went out and it was my friend's birthday, and we just went to a local enough place, and we were kind of a bit nervous going in and it was just really well done everything. It was a big place so there was lots of space, the staff were excellent, like they were not intrusive. But you know you were very clear that you were safe, and they were being safe, and it just felt comfortable and safe,*” (Female, 59, professional/managerial).

On the other hand, the opposite is observed in a hotel restaurant where outdoor seating and tables were so close together customers at different tables were brushing against each other while they had their meal.

*“Last Sunday for instance, I saw a particular hotel and they had tables outside, and there were people sitting at those tables having drinks and food. And I mean, the tables were so close to each other, that one person's back one table was literally touching the back of the person at the other table,”* (Female, 67, retired).

Staff in retail and food service settings described how they could adhere strictly to physical distancing measures with customers but found it more challenging to maintain space between themselves and their colleagues. This was in part due to the nature of the work requiring staff to come into close proximity with each other, but also the “ *mental energy*” needed to remain vigilant about their distancing behaviours being difficult to maintain. *“If it's in front of a customer [physical distancing is] implemented quite well […] but once we close at 11 you have to clean up afterwards. There's no customers so there is no social distancing in place. We're just kind of bumping into each other trying to clean things and get home,”* (Male, 24, student). 

*“We [staff] can do it [maintain physical distance], but there's only so much mental energy you kind of have in a day, and you’re going to have to use that on the customers to be honest,”* (Male, 24, casual worker). 

### Habituation to threat

Living with and adhering to physical distancing measures since March of 2020 meant that the anxiety and worry of the virus has diminished over-time.

***“**I just find I’m not as stressed out about it anymore, I just accept it, (the virus) okay like it's probably going to be fine,”* (Female, 26, unemployed).

There is a sense of desensitisation to the threat of the virus, and so reducing physical distancing.

*“I suppose we know more about the virus and in some ways, the urgency has gone out of it, the panic,”* (Male, 25, student).

Participants also described the potential to *“let our guard slip”* and not adhere to physical distancing guidelines when meeting up with friends.

*“I do meet up with certain friends that I would see often enough and my bandmates to be included in that, like we do kind of let our guard slip the odd time and I think that's almost like human nature as well,*” (Male, 28, professional/managerial).

The perceived threat of visiting friends and family in different households has lessened over time, due to learning about these threats and the potential consequences to other family members but not actually encountering them in our personal lives. Invitations from others suggests they feel the risk is low and repeatedly saying no becomes tiresome.

*“It's like if someone invites you over and you’re like ‘oh yeah’ but then they say, ‘oh mam or dad have an underlying health condition,’ and then it’s like well, why are you inviting me over? I don't know what to do, and then the parents are kind of like, ‘ah no it's okay,’ and then you’re kind of feeling nearly, like I could just say no, which I did initially, but like after a while your kind of like, ah I'll come in for a cup of tea,”* (Male, 23, student).

Participants felt that the distancing posters and floor signs were no longer as salient as they had been in place for so long. The newness and significance of the bright yellow signs and floor arrows has worn off. The more participants are presented with these repetitive public health messages, the more we get used to them and choose not to react to them.

*“It's almost like the yellow ‘X’s on the floor have gotten a bit grubby, so it must be over,”* (Female, 43, working professional).

Participants spoke about their experience of distancing at work, how their thoughts about physical distancing had changed over-time, where they have allowed themselves to become more complacent because the vigilance required to practice distancing every day at work with customers and staff is mentally draining.

“Oh at the beginning it was more like stay the f**k away from me please, it was very, very odd. But now I've kind of relaxed a bit more because I'm just kind of emotionally worn down,” (Male, 24, casual worker).

Being unable to maintain a high level of vigilance over long periods of time reflected this habituation to threat.

*“I think at the beginning, very beginning I was probably adhering to it a bit more (physical distancing), in that I was really making sure to stand well back. I think now I've been doing less than two metres, definitely,”* (Female, 23, casual worker).

### Taking risks to protect well-being

Some participants described how they actively weighed up the risks of (not) physically distancing at times in order to support their own well-being. This need to maintain or enhance their well-being was at times a barrier to practicing physical distancing.

Despite being considered high risk this participant explains how refereeing sport is one area of her life where she was willing to take the risk to not always be in a position to physical distance from others, due to the mental and emotional contentment this brings her.

*“I call this the one area of my life [refereeing sport] that I kind of give myself a little bit of an extra lead, being able to I suppose interact a bit more, and I suppose put myself in scenarios where distancing might not be as possible as other areas, but the payoffs of it for me mentally is huge,”* (Female, 27, professional/managerial).

Participants described that gratification from meeting and chatting with others in a social setting, that need for connection and sense of fulfilment from social interaction was reported to be worth the risk.

“With friends, it's nearly impossible. It literally doesn't [happen], like when we’re indoors we all sit on the couch like doing stupid sh**, like all you can kind of do is limit the amount of people there but at the end of the day it's as much as you do really,” (Male, 25, student).

The choice elderly people made to leave their homes for light exercise at quieter times to protect their mental health during the initial lockdown.

*“The whole business the whole, the early lockdown, I did find it very difficult, I found it mentally difficult, and I certainly was going to get out early in the morning for a walk and late at night for a walk, walking in places where there was hardly anybody else, but I had to do that,”* (Male, 78, retired).

### Personal responsibility to control the “controllables”

Personal responsibility narratives were present in all interviews. Participants felt that having a sense of personal responsibility for their own behaviour was an important enabler of physical distancing. They believed it was their own personal choice to maintain physical distance from others, not only to protect themselves but to protect other people around them.

*“…you know, you have no idea what anyone else is living with, so just be kind and give them their space. If you end up in their space because of a conversation of whatever, well just measure that in terms of is this a risk for you or I? You know, maybe it could be a risk for them because, you know for yourself whether it is or it isn't, and increase the space if need be. Just say, ‘sorry, we'll just keep the two metres and that's for you and for me,’”* (Female, 48, working professional). 

Participants discussed how focusing on that which they could control, specifically their own behaviour, was an adaptive way of coping with the impact of pandemic as well as a facilitator of adherence to physical distancing measures. A female participant felt that once she was adhering to the public health advice by practising the proper hygiene etiquette, wearing a mask, and keeping her distance, she was controlling the situation as best she could to keep herself and her family safe. By allowing herself to control what she could control in terms of her own behaviour, she felt less anxious about the virus getting into her home and affecting her life.

*“I'm very much [for] control of the controllables, and it's probably a way of controlling the situation, for me has been taking the precautions that I believe are best. In terms of using the sanitizer, wearing my mask and keeping distance, and being really conscious of not mixing with big groups of people or lots of small groups of people within a short space of time. I suppose that's reduced the worry for me, because I actually don't know what else I can be doing,”* (Female, 43, working professional).

Aside from responsibility to adhere to physical distancing to protect themselves and friends and family, a sense of responsibility to protect others in the community was also expressed in most interviews. A male student explains how he felt a sense of personal responsibility to maintain physical distance from others out of respect for his landlady’s health and her concern about the virus.

*“I live with the landlady, like the owner of the house here, and I respect her too. Even recently, now, she's just asked me can I stay in, you know, until we just see what happens. I'd have to respect it for her too because it's her home. She wants to feel safe here,”* (Male, 23, student).

### Confusion and uncertainty around government guidelines

Confusion and uncertainty about government guidelines was perceived to be a barrier of physical distancing. There were two aspects to this confusion: 1) what the guidelines actually are and 2) the rationale for different rules. There was also some concern over unclear guidance around physical distancing in certain settings, and not being able to always maintain the gold standard of two metres. 

*“But with the opening of barbers they were saying like it needs to be one metre. So if it's one metre or if it’s two metres, I think it's probably two metres is probably the gold standard. But I think that communication wasn't very clear at all,”* (Male, 25, student)

Participants felt the uncertainty around how many households or people could visit another household at Level 3 was confusing and created challenges to abide by physical distancing guidelines. At this time government guidance was changing from week to week in early September through to October and modifications to different levels were also being introduced. For example, Dublin and Donegal moved to Level 3 mid-September, and all other counties on 6 ^th^ October 2020. Cavan, Monaghan and Donegal then moved to Level 4 on 15 ^th^ October ( Government of Ireland's plan for living with COVID-19). These moves to different levels impacted the number of household visitors that were allowed, so as this participant points out, it was difficult to follow.

*“So, we have the five levels and then there’s the rules on the household, and how many houses can visit and how many people? I mean, I've no idea, I have no idea we’re at Level 3 now and I don't know how many households and how many people, I think that kind of made it a bit difficult, I think that kind of confuses things an awful lot for things like socially distancing,”* (Female, 26, unemployed).

Similar confusion around the rational for these rules was apparent, for example, why at Level 3 people could socialise outside in a large group, in a restaurant or bar setting that serves food but were not permitted to have anyone visit a private garden and sit outdoors. Participants were unclear of the rationale for this guideline as maintaining distance with a selected few people in a garden was seen as easier than maintaining distance when seated outdoors at a restaurant with people from many different households.

*“What I couldn't understand was at level three, you couldn't meet anybody in the garden of your own house. But yet you could meet 15, was it 14/15 people outside in a restaurant, or outside in a pub. So, for me, that was crazy. I'm like, more than likely meeting people in your own garden, you’re only meeting a few you might not meet 15. But I honestly couldn't understand the logic behind that,”* (Female, 67, retired).

## Discussion

The current findings identify a number of key internal and external psychosocial factors that promote or impede adherence to physical distancing. The main themes identified were: (1) Maintaining and negotiating close relationships; (2) Public environments support or discourage physical distancing; (3) Habituation to threat; (4) Taking risks to protect well-being; (5) Personal responsibility to control the “controllables”; and (6) Confusion and uncertainty around government guidelines. These findings are reflective of a period in time (September – October 2020) when restrictions in Ireland were more relaxed in comparison to a more severe lockdown scenario which went before (Wave 1) and came after (Wave 2). Maintaining and negotiating close relationships; the abnormal feeling of having to distance from family and negotiate distancing when meeting up with friends was a key barrier to this public health measure. All individuals have a basic need for human contact and the strangeness of engaging in uncharacteristic behaviours such as avoiding tactile gestures was focused on in some capacity across all interviews, and is further supported by findings from the iCARE Study data ( Durand *et al.*, 2021). Another key barrier was habituation to threat through a process of becoming increasingly familiar to the threat of the virus overtime. This may be due to the gradual lifting of heavy lockdown restrictions from June to September when Ireland was in Phase 2 of the Government’s roadmap for re-opening society and business. At this time residents living in Ireland could travel within their own county or up to 20km from their home, groups of up to 6 people could meet indoors or outdoors, non-essential retail was open, 15 people were permitted to meet outdoors for organised sports training, social or cultural activities, and up to 25 people could attend a funeral and those who were medically vulnerable, and the elderly were allowed a small number of visitors. This relative easing of restrictions may have reduced the anxiety and worry about the potential threat of the virus, and increased complacency around physical distancing behaviours. Data from 1,600 individuals who took part in the Amárach public opinion survey in December 2020 supports this, showing a significant decline in the number of individuals experiencing worry from March (54%) to September (35%) ( Department of Health, 2020–2021). Additionally, participants’ choice to prioritise their mental well-being and engage in activities and hobbies where physical distancing was not always possible was also a barrier. This is consistent with other international research ( Scott *et al.*, 2021). Given the increased uncertainty and prolonged period of strict lockdown measures from March to early June, and the growing concern of a second wave in mid-September, it is only natural that people were going to have difficulty adhering to physical distancing and persevering with these guidelines long term, as maintaining behaviour change over a lengthy period of time is challenging and difficult to achieve ( Kwasnicka *et al.*, 2016).

Public environments that support or discourage physical distancing was widely communicated as both a barrier (e.g., tables being too close to others, overcrowding in shops, rotation of staff and minimal ventilation or partitions) and facilitator (e.g., seating arranged to accommodate physical distancing for customers, online menus, use of partitions and feeling more comfortable and at ease encouraged adherence to distancing). The main factor that promoted adherence for individuals included controlling what they could control; for example, avoiding crowded places, keeping a two-metre distance and wearing a mask. The motivation to carry out these behaviours was to protect themselves and their family, this is in line with findings from the iCARE Study for Ireland which found that concerns about one’s own health and concerns about the health of others was linked to higher rates of adherence to physical distancing measures ( Durand *et al.*, 2021), and is also in line with findings by Lunn *et al.* (2020) which found messages that emphasised the risk of infecting vulnerable people or the risk of infecting large numbers of people resulted in increased motivation from Irish citizens to engage in physical distancing behaviours and greater acceptability of these behaviours. The confusion around Government guidelines on number of household visitors also created challenges in following distancing guidelines. This relates to a finding reported as part of the iCARE Study in Ireland by Durand *et al.* (2021), where one of the Government policies that reported the lowest levels of awareness was for avoiding social gatherings. Regional variations in the level of restrictions, not to mention further modifications to these levels by Government officials resulted in ambiguity which could have been avoided.

The six themes described above represent barriers and facilitators that were most consistent across all 25 interviews. There were, however, other relevant findings evident from the data that warrant discussion. First, though the majority of participants felt they personally adhered to physical distancing measures as frequently as they could, many spoke about perceived non-adherence by others. This was evident across all age groups, particularly in retail settings, which is consistent with findings from Williams *et al.* (2020). Similarly, a recent behavioural study suggests that Irish citizens are largely over-estimating other people’s social activity: 81% believed that they were following the public health mitigation measures better than others, while 25% reported meeting three or more people outside of their household, yet they believed they were meeting fewer people than average ( ESRI, 2021). Interestingly, younger participants described observing a lack of adherence by middle-aged and older groups, while older participants perceived the student population as being least adherent to distancing measures. This coincides with further data reported as part of the iCARE Study in Ireland, which found that young people between the ages of 25–34 years were least adherent to many physical distancing measures including keeping a two-metre distance, avoiding social gatherings, and avoiding non-essential travel ( Durand *et al.*, 2021); however, the differences in adherence between age groups in the iCARE analyses were small. This pandemic has drastically changed and even prevented many developmental milestones such as graduations, and summer internships in the United States (US), which has taken away experiences and opportunities to thrive for young people. Young people have a growing need to interact socially and build relationships with others ( Holmes *et al.*, 2020). Furthermore, all participants were highly critical on their opinions of non-adherence observed by others, perhaps it is the case that people lack trust in other people’s distancing behaviour ( Kaspar, 2020). Second, the challenges of maintaining distancing as an essential worker, specifically working in retail and healthcare settings, caring for older adults and those in disability services was communicated by individual participants working in these settings. There was a sense of worry and anxiety about the risk of contracting COVID-19 at work and spreading the disease to family and friends. This source of worry aligns with Department of Health Amárach research, which reported the health of family and friends to be the greatest source of worry ( Department of Health, 2020–2021). We spoke to individuals with pre-existing health issues which placed them in the high-risk category. These individuals talked about cocooning, not visiting family, and having a housemate or friend do their shopping for them or ordering online. Similar findings were reported by Coroiu *et al.* (2020) and Durand *et al.* (2021), who found that participants with a chronic physical health condition reported avoiding social gatherings, avoiding bars and restaurants and non-essential travel somewhat more frequently than those at lower risk. Finally, when asked what they understood by the term ‘physical distancing’, participants described keeping a two-metre space between themselves and others outside of their household and wearing a mask where possible. Public health officials describe physical distancing as a set of measures intended to prevent the spread of a contagious disease by maintaining a physical distance between people, and reducing the number of times people come into close physical contact with one another ( Harris *et al.*, 2020). While the understanding of physical distancing in our sample is technically correct, not one individual identified the other forms of physical distancing that the public has been asked to adopt. This lack of clarity regarding what physical distancing measures broadly constitute may be a considered a barrier in and of itself.

### Limitations

While our sample was diverse in terms of age, gender, and socioeconomic background, the voices of more marginalised groups in society may not have been captured. Nonetheless, our study included participants who were retired over 70 years of age, which allowed us to explore the barriers and facilitators in older people who may have been most significantly impacted by COVID-19 and who have been advised to stay at home during lockdown, unlike other studies such as Williams *et al.* (2020) who only included participants up to 60 years old. Including at-risk groups like those over 70 was important for our study in the Irish context. Our PPI group assisted with these recruitment efforts to ensure we reached as diverse an age cohort as possible. While some of the participants interviewed were considered high risk due to having underlying health conditions, no one we interviewed had previously tested positive for COVID-19, so we could not explore the experience of quarantine and isolation. This is an area where the psychosocial impact of physical distancing should be explored. Additionally, all participants volunteered to be part of the study which may result in selection bias, as these individuals could potentially have a vested interest in the research topic and hold different views about physical distancing compared to the general public.

Limitations notwithstanding, this study has several important strengths. The use of PPI in planning and implementing this study ensured the research was carried out appropriately, was interesting and relevant to the general public, and provided a comprehensive account of barriers and facilitators relevant to physical distancing behaviour. Involving members of the public in research, particularly from the outset of a study, can greatly improve the quality and richness of the research. Involvement of PPI partners in COVID-19 research is particularly important ( Murphy *et al.*, 2020). In this case, having PPI contributors advise on how to recruit a diverse sample of participants for this research ensured we were able to recruit a sufficiently large sample remarkably quickly, which is a key advantage given the importance of producing high-quality rapid evidence to combat the effects of COVID-19 on our societies. Having PPI contributors involved in conducting the analysis is also a key strength. Involvement in the qualitative data analysis meant that PPI in this study moved from consultation, which is a relatively modest approach to PPI, towards collaboration. Future research may benefit from consistent meaningful involvement of members of the public.

## Conclusion

Our study identified many barriers but few facilitators to physical distancing based on the perceptions and experiences of the Irish public. These factors vary depending on context and lifespan stage. At the time of data collection, when Ireland was heading into its second wave of the pandemic, there was some degree of acceptance for maintaining physical distancing among the public and a motivation to keep following the public health guidelines until a vaccine became available. That said, it is important to note that physical distancing measures are likely to remain a vital element of public health responses to the pandemic for some time to come, despite the ongoing rollout of COVID-19 vaccination programmes. The proportion of the population that must be vaccinated against COVID-19 to achieve herd immunity remains unclear but is likely to be very high given the highly infectious nature of the disease ( World Health Organization, 2020). Issues of vaccine refusal, limited access to vaccinations for people living in low and middle-income countries, and limited opportunities for children to become vaccinated will undoubtedly hinder the immediate impact of mass vaccination programmes. Understanding barriers and facilitators of adherence to physical distancing is therefore critical to ensure efforts to maintain public buy-in to behavioural prevention measures are effective. Considering Irish lockdown restrictions are believed to be among the strictest in Europe ( Hale *et al.*, 2020), and based on our findings, particularly the uncertainty and confusion around government guidelines, government leaders and health officials need to ensure clarity of future public health messaging and provide a clear rationale for restrictions that emphasises natural consequences of adherence behaviour in order to promote long-term adherence to physical distancing measures during this and future pandemics.

## Data availability

### Underlying data

Raw data are not publicly available as the transcripts cannot be sufficiently de-identified by redaction. Data will be made available by reasonable request to the corresponding author (email address: hannah.durand@nuigalway.ie). A request is considered reasonable where the intended use for the data is clearly outlined, and where this intended use does not violate the protection of participants, or present any other valid ethical, privacy, or security concerns.

### Extended data

Open Science Framework: Identifying and addressing psychosocial determinants of adherence to physical distancing guidance during the COVID-19 pandemic.

https://doi.org/10.17605/OSF.IO/ES85A ( Durand *et al.*, 2020b).

This project contains the following extended data:

■ COREQ_Checklist.pdf
■ GRIPP2SF.pdf
■ Physical Distancing Interview Topic Guide v1.pdf

Data are available under the terms of the Creative Commons Attribution 4.0 International license (CC-BY 4.0).
