# Supplementary material for: Exploring barriers and facilitators of physical distancing in the context of the COVID-19 pandemic: a qualitative interview study
Source: HRB Open Res. 2021 Jul 20;4:50. Originally published 2021 May 12. [Version 2] doi: 10.12688/hrbopenres.13295.2 (PMC8385454; doi:10.12688/hrbopenres.13295.2)
Supplement: Peer review history

# Peer review: Reviewer response for version 2

**DOI:** 10.21956/hrbopenres.14555.r29883

**Published:** 2021-08-24

**Authors:** Sara Keller

**Affiliations:** 1 Division of Infectious Diseases, Department of Medicine, Johns Hopkins University School of Medicine, Baltimore, MD, USA

The authors did an excellent job responding to the comments and I would favor the indexing of this manuscript.

Is the work clearly and accurately presented and does it cite the current literature?

Yes

If applicable, is the statistical analysis and its interpretation appropriate?

Not applicable

Are all the source data underlying the results available to ensure full reproducibility?

Yes

Is the study design appropriate and is the work technically sound?

Yes

Are the conclusions drawn adequately supported by the results?

Yes

Are sufficient details of methods and analysis provided to allow replication by others?

Yes

Reviewer Expertise:

Infectious Diseases, healthcare epidemiology, human factors engineering

I confirm that I have read this submission and believe that I have an appropriate level of expertise to confirm that it is of an acceptable scientific standard.

# Peer review: Reviewer response for version 2

**DOI:** 10.21956/hrbopenres.14555.r29882

**Published:** 2021-08-12

**Authors:** Sabahat Ölcer

**Affiliations:** 1 Health Services Research, Faculty of Health, School of Medicine, Witten/Herdecke University, Witten, Germany

This study is remarkable because the findings contribute to understanding how physical distancing measures are perceived and to uncovering barriers and facilitators to adherence to physical distancing measures caused by the COVID-19 pandemic in the day-to-day experiences of individuals. Considering the entire article, a framework for the COVID-19-related physical distancing measures in Ireland is presented. Changes and additions to the first version, in line with the recommendations of the reviewers, have further strengthened all sections. I believe that this study will contribute to the gap existing in the literature regarding the COVID-19-related physical distancing measures in Ireland.

Is the work clearly and accurately presented and does it cite the current literature?

Partly

If applicable, is the statistical analysis and its interpretation appropriate?

Not applicable

Are all the source data underlying the results available to ensure full reproducibility?

Yes

Is the study design appropriate and is the work technically sound?

Yes

Are the conclusions drawn adequately supported by the results?

Yes

Are sufficient details of methods and analysis provided to allow replication by others?

Yes

Reviewer Expertise:

Immigrants; forced migration; emotions; family relations; gender studies; health research

I confirm that I have read this submission and believe that I have an appropriate level of expertise to confirm that it is of an acceptable scientific standard.

# Peer review: Reviewer response for version 1

**DOI:** 10.21956/hrbopenres.14471.r29735

**Published:** 2021-07-06

**Authors:** Sara Keller

**Affiliations:** 1 Division of Infectious Diseases, Department of Medicine, Johns Hopkins University School of Medicine, Baltimore, MD, USA

**Abstract:**

- "Our study found that physical distancing measures are judged to be more or less difficult based on a number of internal and external psychosocial factors, including maintaining and negotiating close relationships, habituation to threat, risk compensation, structure of public environments, personal responsibility, and confusion or uncertainty around government guidelines." This seems a bit diffuse. Which of these make PD more difficult vs less difficult?
- Also, please explain in the results briefly any distinctions as these seem to be a major part of the conclusion.

**Introduction:**

- Physical distancing also has to do with duration of contact--mention this as well.
- Could you describe what "high levels of adherence" means?

**Methods:**

- The study team is to be commended for incorporating community members into their research strategy and analysis despite the need for a rapid turn around of qualitative data results.
- What do you mean by "diverse in terms of age, gender, and occupational category"? Front-line workers vs non front line? Retired vs currently working? It's said in the results but it would be helpful to list the categories in the methods as well.
- Were any inducement provided to the participants (money, gift cards, etc.)? What about the PPI members?
- Thank you for referring to the government's Level 1-5 guidelines. Could the authors also briefly explain the guidelines as they were at the time of the interviews, as they are quite long, and as they may change by the time this paper is published? (For example, Level 1 restrictions were updated November 27 2020, after the interviews were performed; Level 5 restrictions were updated May 4, 2021). A basic "least restrictive" to "most restrictive" would likely be adequate (Ironically, this is a theme identified in this manuscript).

**Results:**

- In terms of occupation, the sort of work being done also likely impacts the responses to questions. Were there any front-line or essential workers (healthcare workers, police, fire fighters, etc. as well as clerks etc.) who had direct contact with the public? Were there any essential workers who had to continue to work in person (perhaps with less contact with the public)? Were people working remotely vs in person?

**Discussion:**

- It might be helpful to take the work further in the conclusion. As community members become vaccinated, what additional guidance could we draw from this study? Importance to those who cannot be vaccinated or may have an inadequate response? Importance to future pandemics? Importance to viral respiratory seasons?

Overall, a very interesting manuscript.

Is the work clearly and accurately presented and does it cite the current literature?

Yes

If applicable, is the statistical analysis and its interpretation appropriate?

Not applicable

Are all the source data underlying the results available to ensure full reproducibility?

Yes

Is the study design appropriate and is the work technically sound?

Yes

Are the conclusions drawn adequately supported by the results?

Yes

Are sufficient details of methods and analysis provided to allow replication by others?

Yes

Reviewer Expertise:

Infectious Diseases, healthcare epidemiology, human factors engineering

I confirm that I have read this submission and believe that I have an appropriate level of expertise to confirm that it is of an acceptable scientific standard, however I have significant reservations, as outlined above.

# Response

**Published:** 2021-07-13

**Authors:** Hannah Durand

**Affiliations:** University of Stirling, UK

Abstract:

- "Our study found that physical distancing measures are judged to be more or less difficult based on a number of internal and external psychosocial factors, including maintaining and negotiating close relationships, habituation to threat, risk compensation, structure of public environments, personal responsibility, and confusion or uncertainty around government guidelines." This seems a bit diffuse. Which of these make PD more difficult vs less difficult?

***Response:* This has been revised within the Abstract.**

- Also, please explain in the results briefly any distinctions as these seem to be a major part of the conclusion.

***Response:* This has been revised within the Abstract.**

 Introduction:

- Physical distancing also has to do with duration of contact--mention this as well.

***Response:* This has been added to the Introduction.**

- Could you describe what "high levels of adherence" means?

***Response:* Here we refer to self-reported frequency of adherence to any physical distancing behaviour operationalised in the original source publications referenced (e.g., maintaining 2-metre distance between oneself and others, avoiding social gatherings, avoiding non-essential travel, etc.). This detail has been added to the Introduction.**

 Methods:

- The study team is to be commended for incorporating community members into their research strategy and analysis despite the need for a rapid turn around of qualitative data results.

***Response:* Thank you for acknowledging this important aspect of our work.**

- What do you mean by "diverse in terms of age, gender, and occupational category"? Front-line workers vs non front line? Retired vs currently working? It's said in the results but it would be helpful to list the categories in the methods as well.

***Response:* This detail has been added to the Methods section under the ‘Sample selection and recruitment’ heading.**

- Were any inducement provided to the participants (money, gift cards, etc.)? What about the PPI members?

***Response:* Participants and PPI members each received a €25 multi-store voucher for their involvement in this study. This information has been added to the manuscript under ‘Sample size and recruitment’ and ‘Patient and public involvement’ headings within the Methods section.**

- Thank you for referring to the government's Level 1-5 guidelines. Could the authors also briefly explain the guidelines as they were at the time of the interviews, as they are quite long, and as they may change by the time this paper is published? (For example, Level 1 restrictions were updated November 27 2020, after the interviews were performed; Level 5 restrictions were updated May 4, 2021). A basic "least restrictive" to "most restrictive" would likely be adequate (Ironically, this is a theme identified in this manuscript).

***Response: *This information has been added to the Methods under the ‘Context’ heading.**

 Results:

- In terms of occupation, the sort of work being done also likely impacts the responses to questions. Were there any front-line or essential workers (healthcare workers, police, fire fighters, etc. as well as clerks etc.) who had direct contact with the public? Were there any essential workers who had to continue to work in person (perhaps with less contact with the public)? Were people working remotely vs in person?

***Response:* The main essential workers interviewed in this study were food service / essential retail workers, who would have had some contact with the public at the time of the interviews. Most of these participants were also students and considered being a student to be their primary occupation. The theme ‘Public environments support or discourage physical distancing’ captures challenges of distancing for both patrons and staff, which has been more clearly emphasised now within the Results section of the manuscript. The majority of participants were able to work from home at least some of the time. This detail has also been added to the Results section.**

 Discussion:

- It might be helpful to take the work further in the conclusion. As community members become vaccinated, what additional guidance could we draw from this study? Importance to those who cannot be vaccinated or may have an inadequate response? Importance to future pandemics? Importance to viral respiratory seasons?

***Response:* We have expanded the Conclusion to reflect broader implications of this research.**

 Overall, a very interesting manuscript.

***Response:* Thank you for your positive and constructive review of our manuscript.**

# Peer review: Reviewer response for version 1

**DOI:** 10.21956/hrbopenres.14471.r29734

**Published:** 2021-07-05

**Authors:** Sabahat Ölcer

**Affiliations:** 1 Health Services Research, Faculty of Health, School of Medicine, Witten/Herdecke University, Witten, Germany

The study needs minor changes for indexing, especially in terms of content, themes, and the objectives and research questions of the study.

1. In the Introduction section and at the end of the second paragraph, “...to reduce the risk of catching coronavirus, such as staying...”, “SARS-CoV-2” or “the virus” can be written instead of “coronavirus.”
2. The Results section involves more of the emotions caused by physical distancing. Hence, the connection between physical distancing and emotions can be briefly mentioned in the Introduction section.
3. The objectives and research questions of the study should be presented in detail.
4. Following the theme of “Having to negotiate distancing with others”, the paragraph starting with “The worry and anxiety of...”: In line with the explanation and quotation used here, a subtitle about emotional ambivalence can be used.
5. “Habituation to threat” can be reworded as “From fear to mediocrity”.
6. “Taking risks to maintain well-being” can be reworded as “Taking risks to protect mental well-being.”.
7. “Personal responsibility to control the “controllables””: The theme can be expressed more clearly because the meaning is very intertwined.

Is the work clearly and accurately presented and does it cite the current literature?

Partly

If applicable, is the statistical analysis and its interpretation appropriate?

Not applicable

Are all the source data underlying the results available to ensure full reproducibility?

Yes

Is the study design appropriate and is the work technically sound?

Yes

Are the conclusions drawn adequately supported by the results?

Yes

Are sufficient details of methods and analysis provided to allow replication by others?

Yes

Reviewer Expertise:

Immigrants; forced migration; emotions; family relations; gender studies; health research

I confirm that I have read this submission and believe that I have an appropriate level of expertise to confirm that it is of an acceptable scientific standard, however I have significant reservations, as outlined above.

# Response

**Published:** 2021-07-13

**Authors:** Hannah Durand

**Affiliations:** University of Stirling, UK

The study needs minor changes for indexing, especially in terms of content, themes, and the objectives and research questions of the study.

- In the Introduction section and at the end of the second paragraph, “...to reduce the risk of catching coronavirus, such as staying...”, “SARS-CoV-2” or “the virus” can be written instead of “coronavirus.”

***Response:* This has been revised in the Introduction.**

- The Results section involves more of the emotions caused by physical distancing. Hence, the connection between physical distancing and emotions can be briefly mentioned in the Introduction section.

***Response:* Emotional factors have been added to the list of theoretical factors involved in understanding adherence to physical distancing in the Introduction.**

- The objectives and research questions of the study should be presented in detail.

***Response: *The aim of this study has been stated more clearly in the Methods section.**

- Following the theme of “Having to negotiate distancing with others”, the paragraph starting with “The worry and anxiety of...”: In line with the explanation and quotation used here, a subtitle about emotional ambivalence can be used.

***Response:* We do not think it is appropriate to add this as a sub-subtheme as has been suggested, as we do not believe that this captures a theme or sub-theme as we understand it. We have however now added the following sentence to that paragraph to recognise the role of emotional ambivalence: “ *Participants expressed ambivalence around negotiating distancing with others, whereby they believed distancing was important but were concerned about potential negative responses from others*.”**

- “Habituation to threat” can be reworded as “From fear to mediocrity”.

***Response: *We thank the reviewer for this suggestion but we do not believe this suggested title for the theme accurately captures the essence of this theme. Therefore, we will retain the current theme title. **

- “Taking risks to maintain well-being” can be reworded as “Taking risks to protect mental well-being.”

***Response:* We have made this change in the manuscript. **

- “Personal responsibility to control the “controllables””: The theme can be expressed more clearly because the meaning is very intertwined.

***Response:* We have revised the section on Personal responsibility to control the “controllables” to be more clear.**
